# Editorial to the special issue *Implicit Serial Learning*

**DOI:** 10.2478/v10053-008-0104-2

**Published:** 2012-05-21

**Authors:** Elger L. Abrahamse

**Affiliations:** Department of Experimental Psychology, Ghent University, Belgium

## 

“Purposeful behavior typically exists from (implicitly and explicitly) learned series of events.” This is a typical justification for research on serial learning. Indeed, it seems to be a sound and legitimate endeavourto try and probe the cognitive and neural foundations of serial learning - because life would be much more effortful without this ability. For example, think about parking your car, playing Bach’s Goldberg Variations on the piano, or typing a letter on your computer: These activities all require serial learning. This special issue entitled *Implicit Serial Learning* is entirely devoted to the topic, its major empirical questions, its numerous methodological challenges, and its link to reality.

Serial behavior as a research topic has a long history in experimental psychology. It was once understood as the product of simple reflex chains, in which perceptual feedback that derived from the previous movement triggers the next - and so on and on. In a now classic paper, Lashley (1951) brought (the acquisition of) serial behavior much more into the realm of cognitive psychology, proposing a more plan-based account (see Rosenbaum, Cohen, Jax, Weiss, & Van der Wel, 2007, for a detailed description). Since then several serial learning tasks, concepts, and theories have been introduced and progressively have led to a long list of unanswered or partly answered research questions about how serial behavior is represented in the mind and brain. Notably, over the decades, a large portion of these questions have zoomed in especially on implicit learning: learning that is incidental and the product of which typically resides outside the realm of consciousness. (For more elaborate definitions and characteristics of implicit learning and corresponding discussions, please refer to Frensch & Rünger, 2003, and to Shanks, 2005.) Most of the questions on the representational basis of implicit serial learning can be roughly categorized into three major topics, and this special issue addresses these topics:

1. *What is implicit serial learning, and how can we distinguish implicit learning from its explicit counterpart?*

The concept of *implicit serial learning* is as intriguing as it is problematic. Various issues are unsolved, both theoretically and methodologically. In answering the question on the nature of implicit learning, most approaches determine its features on the basis of often acclaimed distinctions between implicit and explicit learning, such as the former’s relative automaticity and independence of attentional resources. Mong, McCabe, and Clegg (2012), for example, took up the challenge of identifying distinct processes within the process-impurity (with respect to the automatic/controlled distinction) that is typical for (most) tasks. Mong et al. proposed a novel implementation of the process dissociation procedure in serial learning tasks and concluded that both automatic and more controlled learning processes can be identified in incidental serial learning tasks. Additionally, Wierzcho, Gaillard, Asanowicz, and Cleeremans (2012) tried to distill implicit learning effects by employing a highly demanding - and novel type of - dual task setting that impairs explicit learning; and indeed, serial learning is still observed when attentional resources are strongly occupied by a secondary task. The strong focus on implicit learning, however, should neither take away interest from its explicit counterpart, nor from our looking for novel differences between implicit and explicit learning. Dale, Duran, and Morehead (2012) showed that predictive behavior emerges very early in serial production, but that its development across training strongly co-varies with explicit recall of the underlying regularity - and therefore is mainly characteristic of explicit learning. Schwager, Rünger, Gaschler, and Frensch (2012) contrasted two theoretical accounts for the development of explicit knowledge in an incidental learning task: gradually increasing representation strength and the observation of unexpected events that trigger an intentional search. Their results supported the unexpected-event hypothesis.

2. *What type of information is implicit serial learning based on?*

There is growing consensus that serial learning can rely on both perceptual and response-based regularities. Which is dominant probably depends on the specific learning conditions. This means that we have to understand these conditions. Kirsch and Hoffmann (2012) contribute to this understanding: They explored the effect of manipulating spatial stimulus configuration and observed that such seemingly unimportant task features can modulate the balance between perceptual and response-based learning. Abrahamse, Van der Lubbe, Verwey, Szumska, and Jaśkowski (2012) tried to exploit the accepted notion that implicit learning can be perceptual in nature, and, inspired by the typical perceptual richness of everyday life, explored the effect of the availability of multiple congruent sources of stimulation on implicit learning. This sensory redundancy did not benefit learning.

3. *What type of general learning process underlies implicit serial learning?*

Several general learning mechanisms can be proposed to be at the heart of implicit serial learning. To name a few, consider (a) the formation of associations between successive items, (b) the formation of associations between an item and its position within the full “list” of items (i.e., ordinal structure learning), (c) the formation of a representation of specific series of successive items (i.e., fragmentation or chunking), and (d) the formation of an abstract rule. To complicate matters, implicit serial learning may comprise multiple mechanisms, possibly depending on the specific learning conditions. Both the studies of Franco and Destrebecqz (2012) and of Schuck, Gaschler, and Frensch (2012) showed the latter to be indeed the case. Together they found support for the existence of options (a), (b) and (c), depending on task characteristics (e.g., the saliency of fragments) and the actual moment in training (e.g., early vs. late).

Against the background of these complex questions, ensuring effective and valuable progression of theory on serial learning needs continuous and careful contemplation on the research in this domain and its key challenges. First, at a regular basis, researchers in the field need to summarize and review findings on a specific topic and from different laboratories in order to maintain an overview and to spur theoretical progress. The current special issue offers two such high quality reviews: Gheysen and Fias (2012) discuss and evaluate how to best dissociate and characterize different serial learning systems in the brain, whereas Schwarb and Schumacher (2012) outline how they believe that response selection determines the locus of serial learning in general - thereby integrating a set of seemingly conflictive findings in the literature. Both these reviews offer a broad approach to serial learning, which brings about a refreshing link to other concepts and theories. Second, the field needs to guard its link to reality. The serial learning paradigm can nicely be defended from an applied point of view, but questions that are being pursued now seem to arise mostly from the experimental paradigm - and are no longer inspired by the real world. This concern at first may seem odd - in fact, there are real brains out there in real laboratories with real computers and keyboards, and these brains are really learning new skills during our experiments. What else could we wish for? The answer is - of course - generalizability of findings to (more) natural situations and everyday behavior. In this sense, papers such as that of Norman and Price (2012) cannot be applauded enough. They make the intriguing claim that detecting and utilizing regularity in social interaction - “out there” - may be more implicit than one would estimate from lab experiments, possibly due to the overall greater complexity and richness of stimulus inputs and action repertoires in the real world. Finally, it is necessary to remain aware of the methodological challenges, and not to bury these out of desperation. In the current special issue, the interested reader can find a multitude of (novel) methodologies that aim to tackle the most complicated issues in (implicit) sequence learning. How can we disentangle implicit and explicit influences on performance (Mong et al., 2012)? Should we mind differences in baseline reaction times when we compare different training and/or test conditions (Abrahamse et al., 2012)? What task should we use as a secondary task in order to best manipulate attentional resources (Wierzcho et al., 2012)?

This special issue, I believe, will be a valuable contribution to the field of serial learning, both through its theoretical and methodological advancements. From my position as guest editor, I would like to thank all the authors and reviewers for their wonderful contributions to this special issue. However, without wanting to divert attention from these, there were some particularly sad events during the realization of this special issue that need to be mentioned here - in a sense they overshadow this special issue.

First, Prof. Dr. Piotr Jaśkowski, founder and first chief-editor of *Advances in Cognitive Psychology*, passed away last year. Prof. Dr. Jaśkowski (1957-2011) was affiliated with the University of Finance and Management in Warsaw (Poland) from 2003 onwards, and during his career conducted research on a wide variety of topics such as visual attention, temporal order judgment, consciousness, hemispheric asymmetry, and many other (a summary of some of his many contributions can be found in Gut & Dalla Bella, 2011). Personally, I experienced Piotr as a supervisor and a friend during my stay as a researcher at the University of Finance and Management in 2008, and it was then that the idea for a special issue on implicit serial learning first arose. Piotr co-authored our contribution to this issue (Abrahamse et al., 2012).

Second, I would like to take a moment to remember Dr. David McCabe, who passed away in the same year. Dave (1969-2011) was affiliated with Colorado State University (Fort Collins, US) from 2006 onwards, and was a co-author for this special issue (Mong et al., 2012). He was best known for his research on human memory, including topics such as working memory, memory and aging, and many more (a summary of some of his many contributions can be found in Castel, Rhodes, Geraci, Parks, & Logan, 2011). Both Piotr and Dave will be sorely missed by the researchers in their fields, and this special issue is dedicated to them.

## References

[R1] Abrahamse E. L., Van der Lubbe R. H. J., Verwey W. B., Szumska I., Jaśkowski P. (2012). Redundant sensory information does not enhance sequence learning
in the serial RT task.. Advances in Cognitive Psychology.

[R2] Castel A., Rhodes M., Geraci L., Parks C., Logan J. (2011). In memory of David P. McCabe.. Association for Psychological Science Observer.

[R3] Dale R., Duran N. D., Morehead J. R. (2012). Prediction during statistical learning, and implications for the
implicit/explicit divide.. Advances in Cognitive Psychology.

[R4] Franco A., Destrebecqz A. (2012). Chunking or not chunking? How do we find words in artificial
language learning?. Advances in Cognitive Psychology.

[R5] Frensch P. A., Rünger D. (2003). Implicit learning.. Current Directions in Psychological Science.

[R6] Gheysen F., Fias W. (2012). Dissociable neural systems of sequence learning.. Advances in Cognitive Psychology.

[R7] Gut M., Dalla Bella S. (2011). Piotr Jaśkowski (1957-2011): Obituary.. Acta Neurobiologiae Experimentalis.

[R8] Kirsch W., Hoffmann J. (2012). Stimulus dependent modulation of perceptual and motor learning in
a serial reaction time task.. Advances in Cognitive Psychology.

[R9] Lashley K. S. (1951). The problem of serial order in behavior. In L. A. Jeffress (Ed.),
Cerebral mechanisms in behavior (pp. 112-131)..

[R10] Mong H. M., McCabe D. P., Clegg B. A. (2012). Evidence of automatic processing in sequence learning using
process-dissociation.. Advances in Cognitive Psychology.

[R11] Norman E., Price M. C. (2012). Social intuition as a form of implicit learning: Sequences of
body movements are learned less explicitly than letter
sequences.. Advances in Cognitive Psychology.

[R12] Rosenbaum D. A., Cohen R. G., Jax S. A., Weiss D. J., van der Wel R. (2007). The problem of serial order in behavior: Lashley’s
legacy.. Human Movement Science.

[R13] Schuck N. W., Gaschler R., Frensch P. A. (2012). Implicit learning of what comes when and where within a sequence:
The time-course of acquiring serial position-item and item-item associations
to represent serial order.. Advances in Cognitive Psychology.

[R14] Schwager S., Rünger D., Gaschler R., Frensch P. A. (2012). Data-driven sequence learning or search: What are the
prerequisites for the generation of explicit sequence
knowledge?. Advances in Cognitive Psychology.

[R15] Schwarb H., Schumacher E. H. (2012). Generalized lessons about sequence learning from the study of the
serial reaction time task.. Advances in Cognitive Psychology.

[R16] Shanks D. R., Lamberts K., Goldstone R. (2005). Implicit learning.. Handbook of cognition.

[R17] Wierzcho M., Gaillard V., Asanowicz D., Cleeremans A. (2012). Manipulating attentional load in sequence learning through Random
Number Generation.. Advances in Cognitive Psychology.

